# What do Consumers Read About Meat? An Analysis of Media Representations of the Meat-environment Relationship Found in Popular Online News Sites in the UK.

**DOI:** 10.1080/17524032.2022.2072929

**Published:** 2022-10-03

**Authors:** Gilly Mroz, James Painter

**Affiliations:** a Department of Zoology, University of Oxford, Oxford, UK; b Reuters Institute for the Study of Journalism, University of Oxford, Oxford, UK; c School of Geography, University of Oxford, Oxford

**Keywords:** Meat, environment, UK media, low-income groups

## Abstract

Previous scholarship suggests that elite media have tended to pay little attention to the adverse environmental impacts associated with meat consumption and production. Through content analysis of 116 articles from 2019, published on eight popular online news sites consumed by a wide range of demographics in the UK, including lower-income groups (the sector most likely to eat meat), we identify common anti-meat and pro-meat environmental narratives, solutions and recommendations, and the dominant sentiment towards both meat consumption and production. We observed a significantly greater presence of anti-meat consumption and/or production narratives than pro-meat. Over half the articles showed anti-meat consumption sentiment, with only 5% predominately in favour. 10% were against unspecified or industrial production practices, 28% were against industrial-scale farming but supported sustainable methods; and none were entirely in favour of the meat industry. These findings are reflected in the dominant recommendation, present in over 60% of articles, to eat less meat. Our results add substantially to previous media research, particularly showing the increased volume of coverage of the meat-environment nexus, varying levels of contestation around meat eating, and the division of responsibility between consumers and industry.

## Background

Over the past few years a “mainstreaming” of veganism has taken place in western countries (Sexton et al., 2022), with plant-based eating (which focuses on plant-derived foods and either restricts or excludes animal products) increasingly popular (Giraud, 2021). 2021 saw a record number of people (582,538) sign up to participate in Veganuary, an annual campaign which encourages consumers from over 200 countries and territories to adopt a vegan lifestyle throughout January (Veganuary, 2021). In the UK, 9% of adults describe themselves as vegan or vegetarian, according to a 2021 YouGov survey commissioned for this study. In 2020 the UK was the largest market in Europe for plant-based meat alternatives (Smart Protein, 2021). All major British supermarkets have their own vegan ranges (Butler, 2018), while restaurants continue to expand their plant-based offerings and fast-food chains to introduce plant-based alternatives to their meat-based bestsellers (Walker, 2020).

There are various drivers behind the increasing popularity of plant-based diets. Several studies and surveys have found health to be the main reason an omnivore might reduce their meat intake; environmental and animal welfare concerns often appear lower down the list of priorities but play an important supplementary role (Jones (2020), for the UK; Mullee et al. (2017), for Belgium). This suggests that potential individual advantages are a greater driver for dietary change than environmental or ethical benefits (Bryant, 2019); conversely, animal welfare is often cited as the most important reason for meat-elimination by vegans and vegetarians (Jones, 2020).

The meat industry has a clear impact on the environment. Livestock production is a major emitter of greenhouse gases (GHGs), with a common estimate suggesting it contributes 14.5% of all anthropogenic GHG emissions (Gerber et al., 2013). Some research suggests that the level of emissions produced by the sector is so high that, even if fossil fuel emissions were halted immediately, the continuation of current food systems emissions would prevent attempts to limit global warming to the Paris Agreement’s target of 1.5°C, or even 2°C, by the end of the century (Clark et al., 2020). In addition to methane emissions from ruminants and methane and nitrous oxide from manure management (Herrero et al., 2013), high levels of carbon dioxide emissions are largely caused by deforestation and land change to create pasture for livestock and to grow crops for animal feed (Harris et al., 2012), a process which also contributes to biodiversity loss (IPBES, 2019). Although extensive, grass-fed grazing systems can promote carbon sequestration, the estimated benefits are generally modest and often outweighed by livestock-generated emissions (Godfray et al., 2018). Instead, some research suggests that reductions in individual meat consumption can result in significant reductions in GHG emissions (Poore & Nemecek, 2018).

Previous research shows that detailed, science-based reports which highlight the impact of food systems on the environment increase media coverage of the meat-environment nexus (Kristiansen et al., 2021). Two particularly important reports were published in 2019, although their authorship, purpose and focus differed: the Intergovernmental Panel on Climate Change (IPCC)’s Special Report on Climate Change and Land (SRCCL) (Intergovernmental Panel on Climate Change, 2019) and the EAT-Lancet report (Willett et al., 2019). The SRCCL focused on issues around land use, such as agriculture, forestry and other activities, to reduce GHG emissions. It did not recommend one single solution to help combat climate change, but rather various land use suggestions, with dietary change only a minor aspect, and with no specific recommendations on what a healthy diet might look like. In contrast, the EAT-Lancet sought to identify a planetary diet which could help to avoid “both reduced life expectancy and continued environmental degradation” (Willett et al., 2019) (p. 484). The report provided general dietary recommendations, including reducing meat intake to an average of 14g/day for red meat and 29g/day for poultry. While these figures were based mainly on health and nutrition research, studies cited in the report regarding the environmental benefits of vegan and vegetarian diets, as well as of replacing red meat with white, also played a key role in the report’s recommendation for a significant decrease in meat consumption among high-consuming individuals.

These dietary recommendations are particularly relevant for the UK, which in 2019 consumed almost two times more meat per person than the global average (61.45kg/cap versus 34.01kg/cap) (OECD-FAO Agricultural Outlook, 2020). Although beef consumption levels have decreased slightly over the last decade (Public Health England, 2019), poultry intake has increased significantly (OECD-FAO Agricultural Outlook, 2020), probably in relation to its widely accepted (though not uncontested) lower environmental impact and better health profile (Bergeron et al., 2019; LEAP, n.d.). But there are other factors that may explain to some extent high meat consumption levels, both in the UK and globally. First, political institutions might be unwilling to impose restrictions to encourage consumers to reduce their meat intake, such as a meat tax, since the livestock industry plays a key role in many countries’ economies and offers employment opportunities (Godfray et al., 2018). Second, individuals might be reluctant or unable to limit or eliminate meat from their diets. Studies have shown that one of the main barriers to adopting a plant-based diet is individual enjoyment of meat; others include an unwillingness to change habits and routines, the perceived inconvenience and difficulties in preparing plant-based meals, the idea that meat consumption is natural or necessary, and cost (Bryant, 2019; Graça et al., 2015; Lea & Worsley, 2003).

In lower-income countries undergoing rapid urbanisation, the relationship between rising levels of wealth and meat intake has historically been strong (Popkin, 1998), while in many countries meat consumption has traditionally been associated with status and wealth (Happer & Wellesley, 2019). In the UK, however, a 2021 YouGov survey conducted for this study observed higher levels of meat consumption among lower-income communities (see Supplementary Material, Section 1). It found that adults from lower-income (C2DE) groups were more likely both to consume meat and to continue consuming meat at their current intake level, compared with those from higher-income (ABC1) groups. Around 25% of all respondents said they had recently reduced their meat intake, which reflects the increasing popularity of plant-based eating; the figure was, however, slightly higher among higher-income groups.

Despite the increasing popularity of social media as a source of news (Newman, 2020), research shows that mainstream media articles circulate widely and motivate discussion on platforms like Twitter (Sanford et al., 2021). They thus remain a vital source of information that can help to shape not only which issues are discussed, but also in what manner, particularly around the topic of the environment (Happer & Wellesley, 2019). An understanding of how meat is represented in online news sites read by a wide range of socioeconomic demographics can therefore offer an insight into the types of environmental discussions that exist around its consumption and production.

### Media coverage

Various studies have examined media representations of the impact of meat on the environment and/or climate change. This research has mostly focused on elite, legacy print media, with the consensus that the meat-environment nexus is largely underreported across all countries studied.

Several studies focus on the impact of the meat industry on climate change. Neff et al. (2009) found that only 2.4% of their 4,582 climate change articles from 16 US newspapers between September 2005 and January 2008 mentioned the impact of animal agriculture, with only 0.44% substantially focusing on the topic. Kiesel (2010), who examined articles from the Guardian/Observer and the New York Times between November 2006 and December 2008, found that less than 0.13% of climate change articles discussed the topic. More recently Kristiansen et al. (2021) found that only around 4% of climate change articles in two UK and two US newspapers between 2006 and 2018 mentioned the impact of animal agriculture.

Studies that consider meat consumption more generally have found similarly low results. In their study of eight Australian newspapers between 2008 and 2013, Friedlander et al. (2014) found that only 0.96% of their climate change articles also discussed meat. Almiron and Zoppeddu (2015), who looked at articles linking both meat consumption and production to climate change in five Spanish and five Italian newspapers between 2006 and 2013, found that only 1.5% of Spanish and 3.6% of Italian climate change articles mentioned the impact of meat. The authors describe the lack of coverage as a “media blind spot”.

Other studies have looked more generally at meat-related articles. Friedlander et al. (2014), in their study of Australian newspapers, found meat articles appeared most frequently in the themes of animal welfare (25.58%), the economy (24.91%) and food (17.94%), and least frequently in environment (7.97%) and climate change (4.98%). Chiles (2017), in his analysis of 12,664 meat-related New York Times articles between 1983 and 2011, found that the most common themes were cuisine (34.95%) and the economy (32.22%), with the environment (8.71%) ahead only of animal welfare (4.28%) and vegetarian (3.05%). Having interviewed several focus groups, he found that for many participants “meat consumption was an ordinary and routine habit that did not provoke critical reflection” (p. 798).

The acceptance of meat consumption as normal is reflected in the media’s relative silence around its negative environmental impact. Almiron (2020) attributes this silence to the denial of the existence of “the moral anthropocentrism that prevails in society and prevents humans from adopting the important behaviour changes needed to mitigate global warming” (pp. 1–2). Whatever the reason, this silence plays a key role in the lack of awareness in many parts of society concerning the impact of meat on the environment (Happer & Wellesley, 2019).

Despite the large body of literature observing the media’s “normalisation” of meat consumption and production, one study suggests that a softening of attitudes is beginning to take place regarding plant-based eating. Morris (2018), in her analysis of the reporting of the Meat Free Mondays (MFM) campaign in UK newspapers between 2009 and 2015, found that 38% of articles discussed the MFM movement positively, 32% negatively, with the remainder neutral or mixed. Though her results show some polarisation, she observed, in contrast to the previous studies that found an underreporting of meat-climate change stories, that positive attitudes were often framed in relation to the environmental benefits of plant-based eating, and concluded that print media “can be understood as a mechanism that, at least in the UK context, is working in support of de-meatification” (p. 447).

In contrast, Sievert et al. (2021) found more articles to be in favour of meat than against it. In their examination of how various interest groups framed red/processed meat reduction in 150 online news media articles from the US, UK, Australia and New Zealand around the release of four high-profile reports, they categorised 40% of their articles as “pro-meat”, 35% as “pro-reduction”, 21% as “neutral”, and 1% as “Anti-EAT” (for articles focusing on the EAT Foundation rather than red/processed meat). In addition to high levels of polarisation, they found that quotations by academics and nutrition experts usually aligned with the reports’ findings, whereas those by industry representatives were critical of them.

### Research Questions

Although media portrayals of the meat-environment nexus are a growing area of study, there are several areas in which research is lacking. First, with the exception of Sievert et al. (2021), the previous literature does not extend beyond 2018. By looking at articles from 2019, we aim to update and expand this area of scholarship. We are particularly interested in exploring whether the relative silence around the meat-environment relationship remains true, especially during a year in which several reports were published, and whether “de-meatification” is indeed taking place in the British media. Second, previous studies have tended to focus on elite print media usually consumed by higher-income groups. We instead analyse online news media consumed by a broader range of income groups, paying particular attention to tabloid newspapers consumed by lower-income groups and news sites that target more niche audiences and which are seldom included in media studies research. Third, previous scholarship has tended to examine representations of the impact of meat in general, or meat consumption or the meat industry in particular, on the environment. We distinguish between two types of meat-related environment stories—meat consumption and the meat industry—to examine any differences in narratives and sentiment, particularly in relation to consumer versus industry responsibility.

This paper aims to bridge these areas of study and fill these research gaps by examining meat-environment articles in eight online news sites widely consumed by lower-income groups in the UK. We aim to answer the following research questions:

RQ1 What are the dominant anti-/pro-meat narratives around the meat-environment nexus?
RQ2 What are the key solutions, recommendations and advice regarding meat consumption and/or the meat industry?
RQ3 What is the overall sentiment of the articles regarding (a) meat consumption and (b) the meat industry?
RQ4 Are there any differences in how different types of media depict the meat-environment nexus? (e.g. do left-leaning outlets display a more anti-meat sentiment, and right-leaning ones a more pro-meat one?)

## Methods and research design

This study conducted quantitative and qualitative media analysis. Articles from online news sites consumed by a wide range of demographics in the UK, including lower-income groups (according to 2020 YouGov survey data (Newman, 2020)), were analysed. Eight outlets were chosen in total. The first to be selected were the top five news sites accessed by this demographic in the week prior to the survey: BBC, 35%; Guardian, 12%; MailOnline, 11%; Sky News, 9%; and the Sun, 8%. A number of news sites followed at 5%; of these we chose the Mirror, LAD Bible and BuzzFeed. In addition to providing balance to the sample—four newspaper websites and four digital media outlets—the left-wing Mirror was chosen to complement the Guardian and contrast politically with the right-wing Sun and MailOnline, while LAD Bible (targeting mostly young male adults) and BuzzFeed (targeting youth audiences) are alternative online news outlets that have not received significant attention in media research. Together they represent a wide variety of outlets with different political leanings, editorial approaches and target audiences.

Articles published between January and December 2019 were collected. This year was chosen not only to offer a more contemporary snapshot of media representations of the meat-environment relationship, but also because several important reports were released during this period. Following various trials of combinations of search terms, articles were collected using the search term “meat”, as terms such as “beef” and “chicken” retrieved few relevant, and many irrelevant, articles. Although “meat” may limit findings to red meat and overlook some instances of poultry, as well as aquatic animals, dairy and eggs, this is acceptable for the present research given the increased emissions rates of ruminants over non-ruminant animals (Godfray et al., 2018) and the focus of this study on meat, rather than on animal products more generally. The database Factiva was used to collect articles from the online newspapers (Guardian, MailOnline, Mirror, Sun). Articles from the online news outlets (BBC, Sky News, LAD Bible, BuzzFeed) were gathered using the Google News “site:” operator, which displays results from indexed pages from a given website (the search string “meat site:buzzfeed.com”, for example, retrieves articles that refer to meat from the BuzzFeed website).

The two researchers divided the online news sites between them and agreed on inclusion and exclusion criteria to ensure only relevant meat-related articles were collected. Only news, opinion and features articles that referred to meat (whether generic or specific types, such as beef) or plant-based alternatives in four or more lines were retained. Articles that discussed meat only tangentially or did not discuss it at all (such as stories about crime involving meat cleavers) were therefore excluded. Following this method, 947 articles were gathered.

The articles were first categorised into broad topics, identified both deductively, based on previous studies, and inductively, based on recurring themes observed during the initial data collection. Computational topic modelling was then implemented, with various iterations enabling the refinement of the number and content of the topics. Finally, data analysis software NVivo was used to manually categorise the articles into ten topics based on salient themes and keywords present in the headline and first four paragraphs of each article (see Supplementary Material, Section 2). Articles could be categorised into more than one topic.

The researchers undertook coding of the 116 “environment” articles following a detailed codebook (see Supplementary Material, Section 3), generated both deductively and inductively. Several rounds of trial coding were undertaken to iron out disagreements and ensure consistency, with the codebook amended after each round. The final codebook consisted of 37 variables. The Cohen’s Kappa inter-coder reliability score was ≥0.8 (strong agreement) for all but five variables. Three of these scored between 0.7 and 0.8 (substantial agreement). The remaining two scored 0.48, due to only one discrepancy between the coders in a dominant sequence of zero coding (a result often given by Cohen’s Kappa); a simple percentage calculation gave >90% agreement. The coders discussed discrepancies and reached agreed coding across all variables.

The codebook was divided into three main areas of inquiry:

 I. Anti-meat and pro-meat narratives, with environment-related arguments for/against meat consumption and/or the meat industry serving as evidence of narrative presence.
 II. Environment-oriented solutions, recommendations and/or advice, coded if they were normative or prescriptive to consumers, producers or institutions.
III. Sentiment, divided into meat consumption and the meat industry.
  1. Meat consumption: articles were coded as anti-meat if they focused on the negative environmental impact of meat consumption (whether generic or specific types) or the environmental benefits of plant-based diets; pro-meat, if they defended meat consumption or questioned the environmental benefits of plant-based eating; and balanced, if they included both pro-and anti-arguments to a significant degree, or neutral, if they had no stance or contained no narratives.
  2. Meat industry: articles were coded as anti-meat if they focused on the negative environmental impact of the meat industry, whether unspecified animal agriculture systems (a common occurrence in journalism, thanks to its tendency to “simplify complexity” (Kristiansen et al., 2021) (p. 8)) or industrial-scale farming in particular; throughout this article we refer to this category as “anti-meat industry” in general. Articles were coded as anti-industrial/pro-sustainable meat industry if they were against industrial-scale farming but in favour of more sustainable production methods; pro-meat, if they defended unspecified or industrial-scale farming; and balanced, if they included both pro-and anti-arguments to a significant degree, or neutral, if they had no stance or contained no narratives.

## Results

### Level of distribution

947 meat-related articles were distributed across ten categories: plant-based (n = 394, 41.6%), food and cuisine (n = 332, 35.1%), lifestyle (n = 307, 32.4%), animals (n = 155, 16.4%), health and nutrition (n = 128, 13.5%), environment (n = 116, 12.2%), economy and politics (n = 115, 12.1%), food safety (n = 98, 10.3%), farming (n = 49, 5.2%) and labour (n = 13, 1.4%).

### Anti-meat and pro-meat narratives

**Figure 1.**
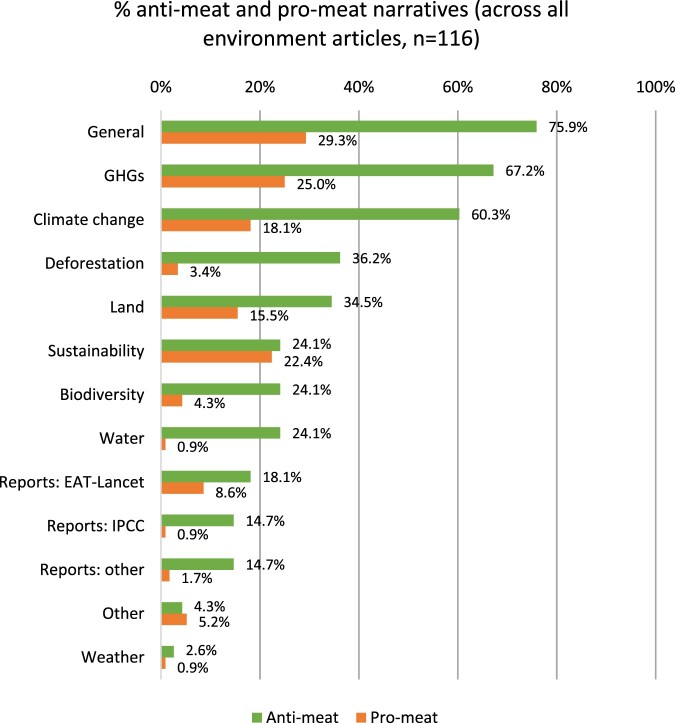
percentage distribution of anti-meat and pro-meat narratives across all environment articles.

Figure 1 shows the percentage distribution of anti-meat and pro-meat narratives across all environment articles (see Supplementary Material, Section 4, for examples). The most common anti-meat narratives were that meat consumption and/or production is generally bad for the environment (75.9%), that meat production leads to high levels of GHG emissions (whether in general, or specifically methane and/or carbon dioxide) (67.2%), and that it is a leading contributor to climate change (60.3%). Deforestation and land use/change were also prominent topics (36.2% and 34.5%, respectively).

There were far fewer pro-meat narratives. Of these, most question or deny the negative impact of meat on the environment, or suggest that certain forms of farming or meats are environmentally friendly (29.3%); that certain farming methods do not have a high carbon footprint, or help to sequester carbon (25.0%); and that certain types of farming are sustainable (22.4%).

The distribution of narratives is largely similar across individual outlets (see Supplementary Material, Section 5). The general impact of meat on the environment was in the top two anti-meat narratives for all news sites, and the impact of GHG emissions from meat production in the top three. The impact of meat on climate change was in the top three for six outlets.

Denial of the general environmental impact of meat and of its high carbon footprint were among the top three pro-meat narratives in six news sites. The sustainability of some farming methods was among the top two in half the outlets, while criticism of the EAT-Lancet report was dominant in three.

### Solutions, recommendations and advice

**Figure 2.**
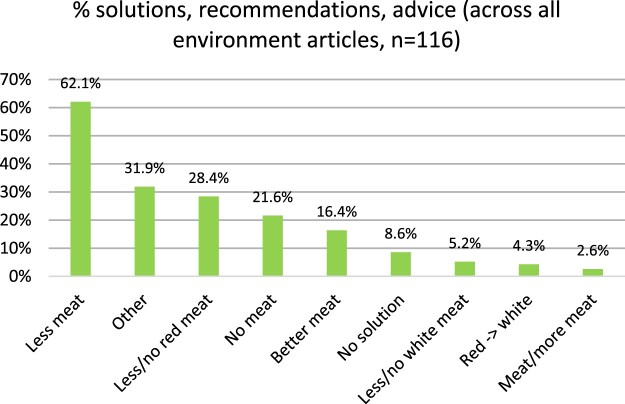
percentage distribution of solutions, recommendations and advice across all environment articles

Figure 2 shows the distribution of solutions, recommendations and advice across all environment articles. The most common solution was to “eat less meat” (62.1%). “Other” solutions followed (31.9%); this category consisted of a variety of solutions that appeared in our sample only occasionally, such as a meat tax, a ban on environmentally damaging foods (including meat), insect-based pet food, and even cannibalism. “Eat less or no red meat” (28.4%), “eat no meat” (21.6%) and switching to “better”, more sustainable types of meat (16.4%) were also popular suggestions.

**Figure 3.**
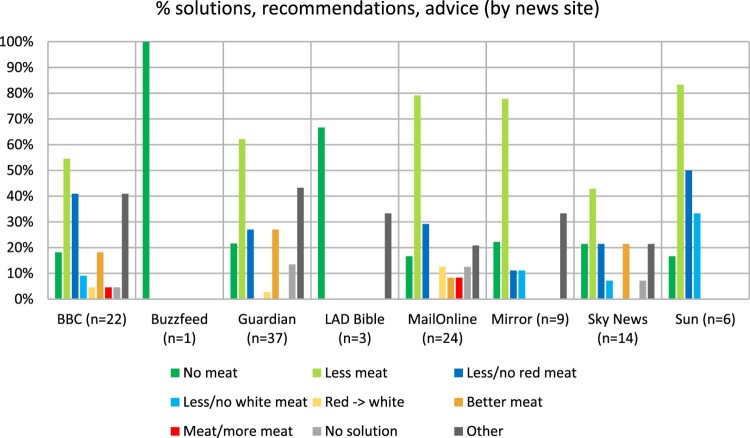
percentage distribution of solutions, recommendations and advice by news site

Solutions were largely consistent across media outlets (see Figure 3). “Eat less meat” was the most common solution in six news sites (Sun, MailOnline, Mirror, Guardian, BBC, Sky News) and “eat no meat” in the remaining two (BuzzFeed and LAD Bible, although their sample was small).

### Sentiment

**Figure 4.**
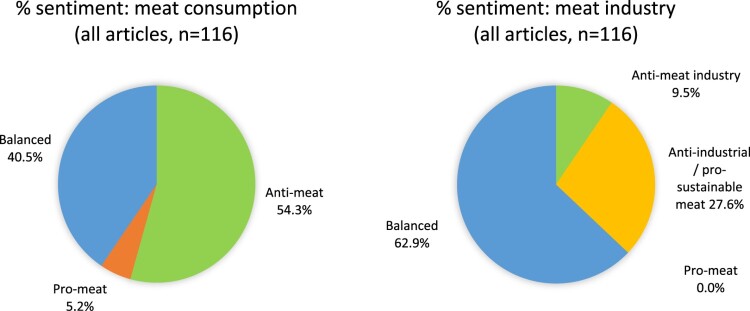
percentage distribution across all environment articles of (a) meat consumption sentiment and (b) meat industry sentiment

Sentiment distribution can be seen in Figure 4. Over half of articles (54.3%) contained anti-meat consumption sentiment. 40.5% were neutral or contained a balance of arguments. Only 5.2% were predominantly in favour of meat consumption or against a shift to plant-based diets for environmental reasons.

Only 9.5% of articles were generally against the meat industry. 27.6% were against industrial-scale farming but in favour of more sustainable methods, as seen, for example, in National Farmers’ Union (NFU) Vice President Stuart Roberts’ assertion that “[…] there is a huge difference between the environmental impact of high-quality British beef for example, versus beef from an animal that is a product of an intensive feed-lot system in a previously bio-diverse area that has been deforested to make way for cattle” (Thomas-Peter, 2019). 62.9% of articles were neutral or balanced. None were in favour of the meat sector, whether in general or in its largely industrial form.

**Figure 5.**
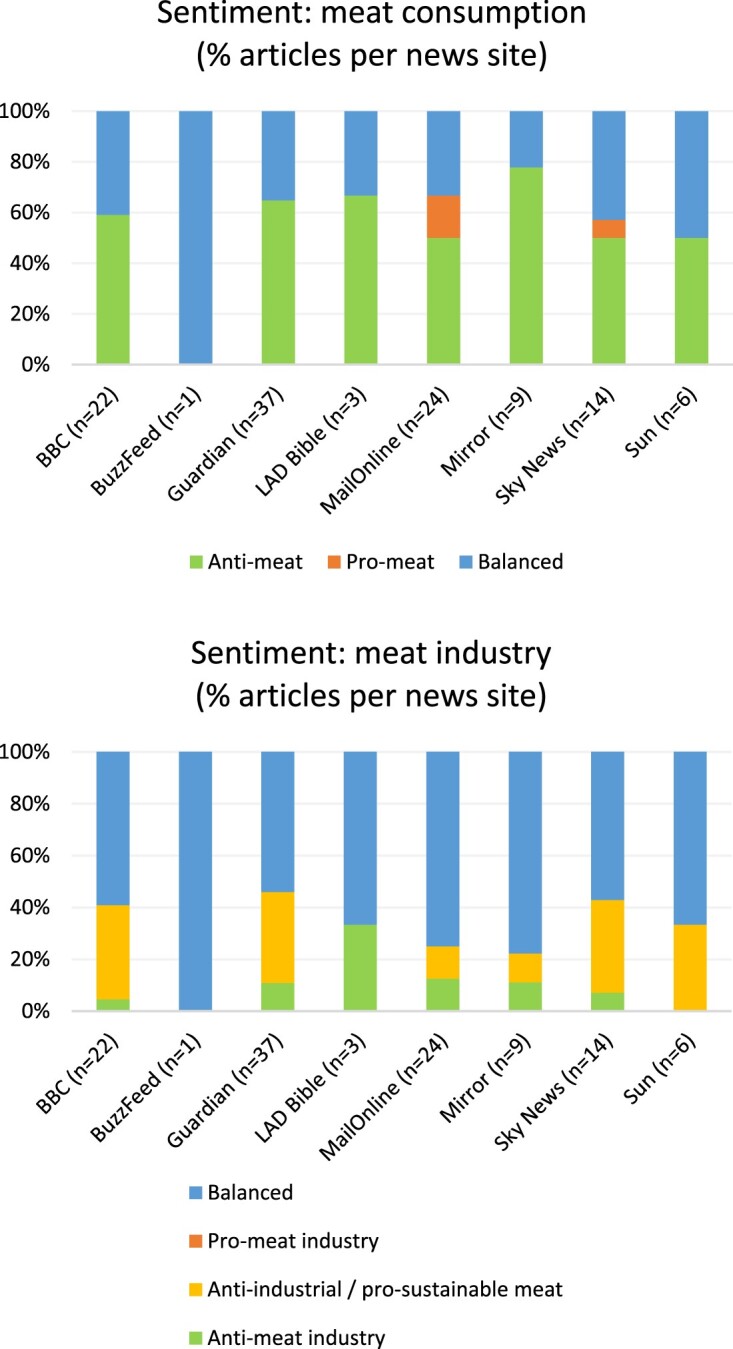
percentage distribution by news site of (a) meat consumption sentiment and (b) meat industry sentiment

Figure 5 shows the percentage distribution of meat consumption and industry sentiment across the individual news outlets (see also Supplementary Material, Section 6). Seven out of eight had anti-meat consumption sentiment in half or more of their articles (BuzzFeed’s one neutral article excepted). The Mirror had the highest proportion of anti-meat consumption articles (77.8%), followed by LAD Bible (66.7%) and Guardian (64.9%). Only two outlets had articles with pro-meat eating sentiment: MailOnline had four (16.7%) and Sky News had one (7.1%).

LAD Bible had the highest proportion of anti-meat industry articles (33.3%), but its sample size was small (n = 3). It was followed by MailOnline (12.5%), Mirror (11.1%) and the Guardian (10.8%). Four outlets had over a third of their articles as against industrial-scale farming, but in favour of sustainable methods: Sky News (35.7%), BBC (36.4%), Guardian (35.1%), and the Sun (33.3%). No news outlets had articles that were entirely in favour of the meat industry.

### Reports: IPCC and EAT-Lancet

Given the importance of the IPCC and EAT-Lancet reports, we also examined their media coverage. Tables 1–2 shows that 14.7% of articles discussed the IPCC report, while 18.1% discussed the EAT-Lancet. Although these figures are not too dissimilar, stark differences appear in the proportion of articles that contain criticism of the reports: only one article criticised the IPCC (5.9% of IPCC articles), whereas almost half (10 articles, 47.6% of EAT-Lancet articles) contained criticism of the EAT-Lancet.

**Table 1.** shows the distribution of articles across the eight news sites. Sky News (32.6%), BBC (28.2%) and Guardian (26.4%) had the highest proportion^1^ of meat-related articles that discussed the environment; the remaining five outlets had fewer than 10% of their articles discussing the meat-environment relationship.

News site	URLs	Total # articles	# environment articles	% environment articles per news site
BBC	bbc.co.uk	78	22	28.2%
BuzzFeed	buzzfeed.com	17	1	5.9%
Guardian	guardian.co.uk	140	37	26.4%
LAD Bible	ladbible.com	74	3	4.1%
MailOnline	dailymail.co.uk	321	24	7.5%
Mirror	mirror.co.uk	105	9	8.6%
Sky News	news.sky.com	43	14	32.6%
Sun	thesun.co.uk	169	6	3.6%
Totals		947	116	–

**Table 2.** Distribution of IPCC and EAT-Lancet articles across all environment articles

Report	# articles discussing the report	% total articles (n=116)	# articles criticising the report	% total articles (n=116)	% report-related articles
IPCC	17	14.7%	1	0.9%	5.9%
EAT-Lancet	21	18.1%	10	8.6%	47.6%

The IPCC report was discussed in five news sites (it was absent from the Mirror, BuzzFeed and LAD Bible). The Guardian had the highest number and proportion of articles discussing the report (8 and 21.6%), including the only article criticising it. The EAT-Lancet was discussed in six outlets (absent from BuzzFeed and LAD Bible). The Sun had the highest proportion of articles that discussed the report (50%, 3 articles), but the Guardian had the highest number (6). All these Sun articles also contained some form of criticism towards it (100%), followed by MailOnline (80%) and the Mirror (50%). This echoes the narratives results (see Supplementary Material, Section 5), with criticism of the EAT-Lancet the dominant anti-meat narrative in the Sun (50%) and joint dominant in MailOnline (16.7%) and the Mirror (11.1%). (For the distribution of report-related articles across individual new sites, see Supplementary Material, Section 7.)

## Discussion

### 1. Differences in media coverage

#### Meat consumption

The media outlets with the highest proportion of anti-meat consumption articles were the Mirror, LAD Bible (albeit with a small sample) and the Guardian. This is perhaps to be expected given the pro-environmental leanings of the Mirror and Guardian (Kristiansen et al., 2021; Painter, 2011). They were followed by the BBC and Sky News (broadcasters that aim for impartiality) and MailOnline and the Sun (right-wing media). The fact that seven of our eight outlets (BuzzFeed excepted) had over 50% of their articles as anti-meat consumption shows a clear trend across all media to focus on the environmental benefits of meat reduction among consumers.

Only MailOnline and Sky News had pro-meat articles. The MailOnline’s four pro-meat articles can probably be associated with its right-wing agenda, while Sky’s one article can perhaps be explained by its aim towards impartiality.

#### Meat industry

With the exception of LAD Bible (n = 3), MailOnline, the Mirror and the Guardian had the highest proportion of anti-meat industry articles. This is perhaps to be expected from the Mirror and Guardian, given their left-leaning editorial stance, which may include a general propensity to be critical of the corporate sector. This is more surprising for the right-wing MailOnline; however, this is offset by its high proportion of balanced articles (75%). In all three cases anti-meat industry sentiment comprised little more than 10% of their meat-environment articles.

Anti-industrial/pro-sustainable agriculture was a far more common sentiment, particularly in Sky News (35.7%), BBC (36.4%), Guardian (35.1%) and the Sun (33.3%). For the first three outlets—two broadcasters that aim for impartiality and a left-wing newspaper—this is probably due to their consideration of the livelihoods of farmers and the economic benefits of sustainable farming. It is somewhat more surprising for the right-wing Sun; however, this is counterbalanced by its being the only news outlet (BuzzFeed excepted) not to feature any anti-meat industry articles.

Given the Mirror’s left-wing and generally pro-environment stance, it is somewhat surprising that it does not contain more anti-industrial/pro-sustainable meat industry articles, with consideration of farmer livelihoods. Instead, this left-wing outlet has the highest proportion of neutral or balanced articles (excluding BuzzFeed). This observation—along with the right-wing MailOnline having the highest proportion of anti-meat industry articles—suggests that while political leanings can play a role, as with the Guardian, this is not universal.

Significantly, no media outlets had pro-industry articles, including the right-wing ones. This contrasts with Sievert et al. (2021), who found that just over 40% of their articles (from the US, UK, Australia and New Zealand) relating to the environment and red/processed meat were in favour of the meat industry; although their figure includes sustainable systems, this is still greater than the 27.6% of articles that were anti-meat industry/pro-sustainable methods in our data. This may suggest a common appreciation across UK media that industrial-scale animal agriculture is harmful to the environment, reflecting the efforts of the British agriculture sector to follow sustainable systems. This is supported by the aforementioned study, which found that meat industry representatives from the UK and New Zealand were particularly well positioned to address environmental concerns (Sievert et al., 2021). Nevertheless, the dominance of neutral or balanced articles across all outlets (over half in all cases) highlights the highly nuanced and contested debate around the environmental benefits and disadvantages of various agricultural practices.

#### IPCC and EAT-Lancet reports

As mentioned, although the total volume of coverage of the IPCC and EAT-Lancet reports was relatively similar, there was a significant difference in how their findings were portrayed, with almost half of the EAT-Lancet articles criticising the report in some way compared with only 6% criticising the IPCC.

Reporting of the IPCC’s SRCCL tended to focus on its suggestion that meat consumption should be reduced to help mitigate global warming, despite it being only a relatively minor aspect of the report. The Guardian had the most coverage, probably a reflection of its pro-environment focus. The report was discussed either positively or neutrally in nearly all articles that mentioned it, across all news sites. One exception was a Guardian article, written by a columnist and not a news reporter, which criticised the SRCCL for “irresponsibly understat[ing] the true carbon cost of our meat and dairy habits” (Monbiot, 2019).

The EAT-Lancet report faced more criticism, exclusively for its dietary recommendations. This was particularly true of right-wing media, with all the report-related articles in the Sun and 80% of those in MailOnline containing criticism. In one Sun article Professor Nigel Scollan, a member of the Meat Advisory Panel, described the report as “‘demonising’ family favourites” (McDermott, 2019), with similar sentiment found in one MailOnline article which described meat eaters being “horrified” by the report’s guidelines, with one Twitter user declaring “life wouldn’t be worth living” on half a rasher of bacon per day (Blott & Spencer, 2019). Only one article, also in MailOnline, drew attention to the issue of affordability, for although the recommended diet “could be cheaper than average for Westerners, more than half of people in sub-Saharan Africa and a third of South Asians may be unable to afford it” (Blanchard, 2019). The left-leaning Mirror and Guardian followed the right-wing outlets in their ratio of critical-to-neutral articles. However, rather than criticising the report’s dietary recommendations, the Mirror tended to direct its criticism towards the hypocrisy of those behind the EAT Forum (which, together with the Lancet journal, convened the EAT-Lancet Commission) for their jet-setting lifestyle (Bagot, 2019) or for “scoffing [a] 20,000-calorie burger” (Shakhnazarova & Carter, 2019). The Mirror is a left-wing, pro-environmental tabloid, which may explain why it accepted the report’s findings, but was less accepting of the failure of the report’s wealthy convenors to practise what they preach to others, particularly to those from less affluent backgrounds. Criticism in the Guardian, conversely, tended to be part of editorial-style articles, embedded among supportive comments that highlighted the impact of current food systems in “driving us towards massive ecosystems damage” (Anthony, 2019). Significantly, none of the articles were critical of the science; rather, they tended to attack what was perceived as a restriction of liberties.

Away from the online newspapers, none of the BBC or Sky News’ coverage of the EAT-Lancet contained contestation, which may reflect the broadcasters’ agenda to report fairly on scientific evidence. However, given the frequent platform they offer to the National Farmers’ Union (NFU), observed below, it is perhaps somewhat surprising that their coverage featured no debate whatsoever.

### 2. Consumer versus industry responsibility

Our results show a clear discrepancy between meat consumption and industry sentiment. Over half of our articles were against meat consumption or in favour of its reduction, compared to just under 10% that were against the meat industry in general. This approximate 5:1 ratio reflects the findings of Kristiansen et al. (2021) in suggesting that the onus is placed far more on the consumer to reduce their meat intake than on the industry to stop producing meat or to switch to plant-based alternatives. This is supported by the dominant solution across our sample: for individuals to eat less meat (a finding also observed by Kristiansen et al. (2021)) in 62% of our articles. It can also be seen in the reporting of the IPCC’s SRCCL, with journalists paying little attention to the focus of the report (discussion of various aspects of land management in relation to climate change mitigation) and instead concentrating on its minor discussion of dietary options.

One reason for this discrepancy is the amount of space afforded to the NFU, particularly by the BBC and Sky News, whose representatives advocate what they see as sustainable farming methods widely implemented in the British meat industry. In one Sky News article the NFU’s Vice President Stuart Roberts emphasised that “the UK meat and dairy farming system is actually one of the most sustainable in the world”, with the “meat is bad, plants are good” conclusion oversimplifying “a tremendously complicated issue” (Thomas-Peter, 2019).

The focus of the NFU away from consumer meat reduction and towards more sustainable animal agriculture is reflected in the second most common solution, “other” (31.9% of articles). A number of the disparate solutions in this category were offered by, or relevant to, the NFU, such as organic farming, improved farming efficiency (such as by using more environmentally-friendly cattle breeds), and subsidies for farmers to switch to more sustainable farming methods. However, they were mentioned less often than ways in which governments and institutions could encourage meat reduction among consumers, such as a meat tax, removing the sale of beef products, or food labels displaying a product’s environmental impact.

The general assignment of responsibility away from the industry and onto the consumer—whether through simple encouragement or stricter policy changes—could, as with the contestation surrounding the EAT-Lancet report, be interpreted by some parties as a restriction of individual liberties, and it is perhaps for this reason that a small number of articles defended the consumer’s right to eat meat. The idea of meat “demonisation”, which appeared in response to the EAT-Lancet report, was also present in these articles, with professor of global agriculture and food security Geoff Simm suggesting that the meat sector was being “demonised” (Sky News, 2019), and another journalist describing beef as “[…] a pawn in the gathering war on meat: a hysterical, ill-informed, one-size-fits-all assault that demonises farmers, butchers and consumers alike. A weapon, if you like, of grass destruction” (Parker Bowles, 2019).

## Conclusion

Our main finding is that, although the overall percentage of articles that make the link between meat and the environment is relatively low (12% of our meat-related articles), those that do often take a critical perspective on meat’s environmental impact, and suggest that some dietary change is necessary, particularly a reduction in meat eating.

To support this statement, and in answer to our specific Research Questions, we found a significantly greater presence of anti-meat consumption and/or production narratives than pro-meat. For example, 76% of articles included the narrative that meat is bad for the environment, compared with 29% that questioned or denied this assertion. Over 60% of articles contained the recommendation to eat less meat, which correlates with our finding that over half the articles demonstrated an anti-meat consumption sentiment, with only 5% in favour. Only 10% of articles were entirely against the meat industry, but almost three times this number were against industrial-scale farming practices but in support of sustainable methods (28%). Significantly, no articles were in favour of the meat sector, either in general or in its current, largely industrial form. There were only minor differences in the reporting of meat-related environment stories among the eight news sites. The left-wing Mirror and Guardian had the highest proportion of anti-meat consumption articles, while the right-wing MailOnline had four pro-meat consumption articles, which may suggest that political leanings do explain some of these minor differences in coverage. This is supported by the high proportions of both anti-meat industry and anti-industrial/pro-sustainable meat production articles in the Guardian. However, the relatively similar proportions of these sentiments in the left-wing Mirror and right-wing MailOnline suggests that, while political leaning can play a role, this is not always the case, especially on issues involving industry and the economy.

We note several other key findings from our analysis. First, we found a higher coverage of meat-related articles that discuss the environment (12.2%) than in previous studies with similar search and categorisational methods (8.71% in Chiles (2017) and 7.97% in Friedlander et al. (2014)). However, these previous studies focused on different countries, time periods and media titles. We do not have a baseline for UK coverage of the meat-environment nexus, but our results would support the view that the UK media pays more attention to this topic than the media in other countries, and that the volume of coverage is probably increasing (Kristiansen et al., 2021), in part driven by the media’s coverage of the IPCC’s SRCCL and the EAT-Lancet reports. At least in the UK, it appears that even though the percentage of meat-related articles which mention the environment remains relatively low, it is difficult to characterise over 100 articles in 2019 as a “media blind spot”. Moreover, the frequent presence of a strong anti-meat consumption sentiment found in our study offers some support to Morris (2018)’s observation of “de-meatification” beginning to take place in the British media, and suggests that environment-related anti-meat consumption narratives are becoming increasingly prominent and normalised.

Second, we found a clear placing of responsibility on the consumer rather than on the meat industry. More articles criticised the individual for eating meat and advocated more plant-based eating patterns than those which criticised the meat industry and/or advocated more sustainable farming methods. This reflects the finding by Kristiansen et al. (2021) of the media assigning responsibility to the consumer more than twice as frequently than to other parties, such as factory farms, farmers, governments or businesses, despite the high (and often undeclared) emissions rates of meat and dairy companies (Lazarus et al., 2021). One reason, the authors argue, for the media focus on individual consumer choice is that it “helps to take the responsibility off the necessary systemic changes that governments should be introducing, and distracts attention from the holding to account of large (polluting) corporations” (p. 14). Our finding is also consistent with the literature which observes that, as part of a neoliberal response to climate change, it is the individual’s responsibility to be a “good” consumer (Sexton, 2018).

Third, in contrast to Sievert et al., (2021), we found little contestation across the different news sites regarding the impact of meat on the environment, including in the coverage of the SRCCL. The exception was the reporting of the EAT-Lancet’s dietary recommendations, which may be explained by the explicit (and, as some perceive, extreme) recommendations for reducing meat consumption. The negative attitudes in some outlets may reflect a phenomenon similar to the “culture wars” found in social media, as observed in the popularity of the “#yes2meat” hashtag on Twitter around the release of the report (Garcia et al., 2019). Sanford et al. (2021) found that meat consumption was one of the most contested topics on Twitter regarding the SRCCL, with tweets containing high levels of toxicity, while Maye et al. (2021) found high levels of contestation on Twitter surrounding environmental concerns and potential solutions, for example in the dominance of “#sustainablemeat” tweets among those involved in meat production versus “#eatlessmeat” among vegan advocates. The low level of contestation in our online news media sample stands out in contrast to that found in social media; however, we would need to supplement our analysis with examination of comments in response to these online articles both “below the line” and on social media. The relative absence of meat-environment contestation also contrasts with the high levels of debate found in health-related meat articles and the lack of consensus in the media (and often among scientists and health professionals) regarding the nutritional benefits and/or harms of meat consumption (Mroz & Painter, forthcoming).

Next, the nuances of the “anti-meat” sentiment present in our sample are significant. We observed an approximate 3:1 ratio both of “eat less meat” versus “eat no meat” recommendations, as well as of articles that displayed anti-industrial meat production sentiment, but were in favour of more sustainable methods, versus those which were against all forms of meat production. The former ratio suggests an openness towards more plant-based ways of eating, as well as the perception of meat reduction as a comparatively more achievable dietary target than veganism or vegetarianism. Moreover, the finding that a fifth of articles included the recommendation to “eat no meat” may serve as evidence for a dampening of the media’s “vegephobia” (Cole & Morgan, 2011). The strong ratio in favour of more sustainable farming methods was particularly stark in the BBC, Sky News and Guardian, and, as suggested above, may be related to their consideration of the socioeconomic livelihoods of farmers. In the case of the broadcasters, it may also be driven by their aim for impartiality; consequently, they offer a large amount of space to representatives of the farming sector (the NFU), who are very vocal in favour of what they see as the distinction between their sustainable farming methods and more industrial-style farming practices (usually from abroad), and critical of the BBC for their perceived lack of impartiality (NFU Online, 2020). However, no articles in our sample—not even from the impartial broadcasters or right-wing outlets—offer positive narratives around industrial meat providers; this may be a reflection in part of the absence of meat industry representatives in the UK willing to be questioned about, and defend, the damage the sector causes the environment.

Finally, as mentioned earlier, lower-income groups in the UK are more likely both to consume meat and to continue consuming meat at their current intake level, but all income groups in the UK could benefit from eating less or better meat for health and environmental reasons (Springmann et al., 2016; Willett et al., 2019). Our results show that by reading popular titles like the BBC, Guardian and MailOnline, they and other social groups are exposed to a significant and regular amount of meat-environment information, with most articles encouraging readers to eat less meat. Although research suggests that information provision alone about the health or environmental consequences of eating meat does not seem to influence dietary behaviour (Bianchi et al., 2018), and that an array of sustained, context-specific interventions is most likely to be successful (Rust et al., 2020), it is difficult to dispute that regular and sustained media coverage at least helps to raise awareness of the impact of diet on health and the environment. However, given the increasingly powerful body of research stressing the clear and urgent need for meat reduction to protect the environment and minimise climate change, the virtual absence of food from such important international meetings as COP26 in Glasgow (Table, 2021), and the lack of sufficient scrutiny of the animal industry by journalists (Khazaal & Almiron, 2016), the onus is clearly on the media to find new and interesting ways to engage readers on this issue, and hold governments and corporations to account.

## Supplementary Material

Supplemental Material

## Note

1 We use the term “proportion” to refer to the percentage of articles containing (a) certain narrative/solution/sentiment(s) out of the total sample from a specific media outlet.
