# Supplementary material for: What do Consumers Read About Meat? An Analysis of Media Representations of the Meat-environment Relationship Found in Popular Online News Sites in the UK
Source: Environ Commun. 2022 Oct 3;17(8):947–64. doi: 10.1080/17524032.2022.2072929 (PMC10721226; doi:10.1080/17524032.2022.2072929)
Supplement: Supplemental Material [file RENC_A_2072929_SM2863.pdf]

Supplementary material

Section 1: YouGov Survey: key data

This survey, commissioned for this study, was conducted in early 2021 to identify consumption habits among different socio-economic groups in the UK. For this reason results were separated by ethnicity, and full national results were not provided. YouGov applied appropriate weighting to obtain these figures.

Which, if any, of these best describes your usual eating habits?	**White (403)**			**BAME (1039)**		
	**ABC1**	**C2DE**	**Total**	**ABC1**	**C2DE**	**Total**
Vegan (do not eat dairy products, eggs, or any other animal product)	4%	0%	2%	2%	1%	2%
Vegetarian (do not eat any meat, poultry, game, fish or shellfish)	8%	6%	7%	8%	5%	7%
Pescatarian (eat fish but do not eat meat or poultry)	2%	2%	1%	7%	5%	6%
Meat eater (eat meat and/or poultry)	69%	77%	74%	58%	70%	62%
Flexitarian (Follow a diet of vegetarian-only days, and then mixed diet the rest of the time)	16%	12%	12%	20%	12%	17%
Other	6%	4%	4%	5%	5%	5%
Don't know	-	1%	0%	0%	2%	1%

Which ONE of the following statements BEST describes how you may be increasing or decreasing the amount of meat you eat?	**White (403)**		**BAME (1039)**	
	**ABC1**	**C2DE**	**ABC1**	**C2DE**
I have recently decreased the amount of meat I eat	24%	22%	28%	25%
I am considering decreasing the amount of meat I eat, but have not done so yet	12%	10%	18%	21%
I am considering increasing the amount of meat I eat, but have not done so yet	-	1%	3%	2%
I have recently increased the amount of meat I eat	1%	2%	6%	3%
Not applicable – I eat meat and don't plan on changing the amount of meat I eat	48%	56%	30%	35%
Not applicable – I don't eat meat and don't plan on changing this	14%	7%	12%	6%
Don't know	1%	2%	3%	8%

Section 2: Topics, themes and keywords

Topic	Themes	Keywords
Food and cuisine	food products, meals, cooking	cuisine, dish, delicacy, menu, meal, snack, breakfast, lunch, dinner, supper, beef, chicken, lamb, pork, sausage, burger, patty, mince, ham, steak, fillet, sandwich, pie, bacon, wrap, nugget, pigs-in-blankets, bolognaise, lasagne, pasta, pizza, roll, meatball, ribs, kebab, burrito, curry, casserole, carbonara, broth, milk, cheese, ice cream, salad, fruit, vegetable, veggie, cook, roast, barbecue, BBQ
Environment	environmental impacts, climate change	environment, climate, global warming, planet, sustainable, unsustainable, emission, methane, carbon, CO2, greenhouse gas, pollution, pollute, pesticide, fertilizer, resources, land, water, deforestation, forest, grassland, soil, crops, biodiversity, drought, fire, bushfire, natural disaster, extreme weather, EAT-Lancet, IPCC, FAO
Animals	animals, living conditions, welfare, rights, activism	animal, welfare, rights, activist, protest, demonstration, campaign, cruel, cruelty, ethical, unethical, abuse, slaughter, rescue, PETA, pet, dog, cat, guinea pig, horse, squirrel, camel, kangaroo, chimpanzee, whale, dolphin, porpoise, seal, fish, crustacean, insect, bee, cow, goat, pig, swine, sheep, lamb, chicken, duck, turkey, bird, avian
Health and nutrition	health, nutrition, dietary impacts	nutrition, nutrient, nutritious, health, unhealthy, deficient, deficiency, immune, immunity, symptom, ill, sick, disease, cancer, cholesterol, osteoporosis, arthritis, diabetes, stroke, blood pressure, hypertension, vascular, Alzheimer's, orthorexia, eyesight, vision, mental health, sexual health, sexual performance, obese, obesity, weight, mineral, vitamin, omega-3, sodium, calcium, zinc, choline, iron, B12, fat, fibre, protein, muscle, heart, bone, skin, body, strong, strength, energy, tired, flu, cold, ailment, death, risk, cure, study, experiment
Food safety	foodborne illnesses, contaminated food, product mislabelling	food safety, hygiene, poisoning, outbreak, foodborne, toxic, toxin, chemical, nitrite, nitrate, chlorine, chlorinated, hormone, antibiotic, contaminated, infect, recall, allergen, superbug, bacteria, virus, parasite, salmonella, E.coli, listeria, campylobacter, cardboard, plastic, rubber, metal, plaster, glass, mould, undercooked, raw, label, mislabelled, horsemeat, scandal
Farming	farming, agriculture	farm, produce, National Farmer's Union (NFU), agriculture, grazing, pasture, crop, grow
Labour	workforce, production, safety	labour, employee, worker, processing plant, meat plant, injury, factory
Economy and politics	economy, trade, politics	economy, politics, policy, deal, tax, trade, sector, supply, negotiation, export, import, invest, price, share, profit, loss, government, law, fine, ban, legal, illegal, EU, European Union, Brexit, council, politician, lawyer, business
Lifestyle	leisure, media, education, travel, celebrities, people	lifestyle, leisure, celebrity, star, actor, actress, singer, broadcaster, presenter, influencer, athlete, bodybuilder, sportsperson, TV, television, programme, documentary, series, film, movie, advert, radio, book, magazine, newspaper, online, social media, Facebook, Twitter, Instagram, YouTube, TikTok, travel, hotel, school, nursery, university, student, pupil, teacher, exam
Plant-based	plant-based, vegan, vegetarian	plant-based, vegan, vegetarian, meat-free, non-meat, meatless, fruitarian, flexitarian

Section 3: Codebook

Narratives (anti-meat)	
1. Environment (general)	Meat is bad for the environment / destroys the planet<br>Eating less meat is beneficial for the environment<br>Meat production/consumption contributes to the destruction of nature<br>Meat production has a high environmental footprint<br>Plant-based diets have a better environmental footprint
2a. Climate change<br><br><br>2b. GHGs<br><br><br><br><br><br>2c. Extreme weather	Discussion of meat production/consumption in the context of CC/global warming (generic)<br>Meat production causes CC/GW<br><br>Meat production produces GHG emissions, which cause CC/GW, e.g.<br>• CO2<br>• Methane<br>Meat production has a high carbon footprint<br>Plant-based diets have a better carbon footprint<br><br>Meat production increases the risk of extreme weather, e.g.<br>• Drought<br>• Fires/bushfires
3. Deforestation	Meat production causes deforestation<br>Meat production causes the destruction/clearance of forests/rainforests/jungles<br>Rainforests are transformed into land for:<br>• Cattle grazing<br>• Soya and crop growth for animal feed
4. Land	Discussion of the impact of the meat industry on land use<br>Discussion of the impact of the meat industry on land change/transformation (generic, no explicit references to deforestation/rainforests)<br>Meat production takes up valuable land for:<br>• Pasture farming<br>• Feed production (crop growth, e.g. soya)<br>It is an inefficient use of land:<br>• Land used to grow crops for animal feed could instead be used to grow crops for human consumption<br>Plant-based diets require less land<br>Meat production (fertilizers, etc.) causes land degradation / soil to lose nutrients
5. Biodiversity	Meat production destroys biodiversity (terrestrial and aquatic) through:<br>• Pesticide/fertilizer use<br>• Land clearance
6. Water	Meat production uses up freshwater<br>Meat production leads to water shortages<br>Meat production contributes to water pollution<br>Fertilizer use causes water pollution (including “dead zones” in the oceans)
7. Sustainability	Meat production is unsustainable<br>Plant-based diets are more sustainable<br>Plant-based food requires fewer resources<br>Meat production is devastating the ecosystem
8a. Reports: EAT-Lancet<br>8b. Reports: IPCC<br>8c. Reports: Other	Presence of EAT-Lancet report<br><br>Presence of IPCC report<br>Presence of other reports (e.g. Committee on Climate Change)
9. Other	Toxic waste from the industry<br>Air pollution from the industry

Counter-narratives (pro-meat)	
10. Environment (general)	Meat production is good for the environment<br>Plant-based diets are also bad for the environment<br>There are other things worse for the environment than meat production<br>There are better ways to protect the environment than reducing meat consumption<br>Giving up meat is not an effective way to help protect/restore the environment<br>Not all types of animal agriculture (e.g. chicken) are bad for the environment<br>Some types of meat (e.g. chicken) are not so bad for the environment<br>Plant-based foods also have a high environmental footprint / not all meat has a high environmental footprint
11a. Climate change<br><br><br><br><br><br>11b. GHGs<br><br><br><br><br><br><br><br><br><br><br><br><br><br><br>11c. Extreme weather	Meat production does not cause CC/global warming<br>Plant-based diets also cause CC/global warming<br>There are other things worse for CC than meat production<br>There are better ways to limit CC/global warming than reducing meat consumption<br>Giving up meat is not an effective wat to help reduce/combat CC<br><br>Meat production is/can be carbon neutral<br>Meat production (e.g. cattle grazing) absorbs/can absorb carbon<br>Certain types of land/tree planting on farms absorbs/can absorb carbon<br>Narratives around carbon sequestration<br>Other industries emit more GHGs than meat production<br>Some meats do not have a high carbon footprint<br>Plant-based foods also have a high carbon footprint / not all meat has a high carbon footprint<br>Methods can be implemented to reduce methane in agriculture, e.g.<br>• Technology can be used to trap methane from cows<br>• Cows can be bred to emit less methane<br>• Cows can be given methane-reducing diets<br>Transportation of non-meat products (e.g. fruits, vegetables) also has a high carbon footprint / emits a lot of GHGs<br><br>Meat production can minimise bad weather (e.g. fewer fires)
12. Deforestation	Crop production (for plant-based consumers) also causes deforestation<br>Intensive livestock farming can help prevent deforestation<br>Responsible farming doesn't necessarily cause deforestation
13. Land	Crop production (for plant-based consumers) also takes up land<br>Some land is only suitable for animal grazing:<br>• Trying to grow crops here would not work/would further impact the environment<br>Cattle farmed on pastures use less land than those farmed on deforested land<br>Land can be farmed in an environmentally friendly way<br>Fertilizers for crop production (for plant-based consumers) cause land pollution/ degradation<br>Land/soil can be restored<br>• *NB: narratives around carbon sequestration fall under 11b (GHGs)*
14. Biodiversity	Meat production improves/can improve biodiversity<br>Crop production (for plant-based consumers) destroys biodiversity, through:<br>• Pesticide use<br>• Land clearance<br>Animals are still killed (whether intentionally or accidentally) in crop production (for plant-based consumers)<br>Some animals are pests/destroy the environment, and it is okay to cull them
15. Water	Crop production (for plant-based consumers) uses up freshwater (e.g. for almonds)<br>Crop production (for plant-based consumers) leads to water shortages<br>Fertilizers for crop production (for plant-based consumers) causes water pollution

	(including "dead zones" in the oceans)
16. Sustainability	Meat production is/can be sustainable<br>Producers can/should move to more sustainable types of farming/meat production<br>Plant-based food still requires lots of resources
17a. Reports: EAT-Lancet<br>17b. Reports: IPCC<br>17c. Reports: Other	Any criticism/scepticism of EAT-Lancet (regarding meat consumption habits or other)<br>Any criticism/scepticism of IPCC (regarding meat consumption habits or other)<br>Any criticism/scepticism of other reports (e.g. Committee on Climate Change)
18. Other	Manure is a good natural fertilizer and can be used as fuel<br>Cannibalism can be effective in preventing global warming<br>Planting trees can help offset environmental issues from animal agriculture (general)<br>• *NB: narratives around planting trees to offset emissions/capture carbon/reduce carbon footprint fall under 11b (GHGs)*<br>• *NB: narratives around planting trees to help mitigate global warming falls under 11a (climate change)*

Solutions, recommendations and advice	
19. Eat no meat	Eat no meat<br>Follow a vegan/plant-based diet
20. Eat less meat	Eat less meat<br>Eat only a little meat
21. Eat less or no red meat	Eat less red meat<br>Eat no red meat
22. Eat less or no white meat	Eat less white meat/poultry<br>Eat no white meat/poultry
23. Replace red meat with white meat	Replace red meat with white meat/poultry<br>Eat less red meat and eat more white meat/poultry
24. Eat better meat	Eat better/high-quality meat<br>Eat grass-fed, free-range meat
25. Continue eating meat	Continue eating meat<br>Eat more meat
26. No solution	-
27. Other	Other solutions, e.g.<br>• Meat tax<br>• Banning meat products<br>• Food labelling with environmental rating<br>• Insect-based food

Sentiment analysis	
A. Meat consumption	-1 Pro-meat:<br>• Against meat-reduction/plant-based diets<br>• In favour of meat consumption<br>0 Neutral or balanced<br>• Neither negative nor positive / no stance / absence of arguments<br>• Pro and con arguments are included to a significant degree (one opposing quote is not enough)<br>1 Anti-meat:<br>• In favour of meat reduction/plant-based diets<br>• Against meat consumption
B. Meat industry	-1 Pro-meat industry:<br>• In favour of the meat industry (all types / generic / unspecified)<br>• In favour of industrial-scale animal agriculture<br>0 Neutral or balanced:<br>• Neither negative nor positive / no stance / absence of arguments

	• Pro and con arguments are included to a significant degree (one opposing quote is not enough)<br>1 Anti-meat industry:<br>• (i) Against the meat industry (generic / unspecified / industrial-scale farming)<br>• (ii) Against industrial-scale farming BUT in favour of sustainable farming (e.g. grass-fed, pasture raised)

Section 4: Examples of narratives and counter-narratives

	**Narratives**	**Counter-narratives**
General	"A vegan diet is probably the single biggest way to reduce your impact on planet Earth […]" (LAD Bible, 1 June 2019)	"Diets with a meaty meal a day are better for the environment than vegetarianism." (MailOnline, 17 September 2019)
Climate change	"Human beings will have to eat less meat to tackle climate change […]" (Mirror, 30 April 2019)	"Meat and farmed animals are wrongly blamed for the climate crisis […]" (Guardian, 15 October 2019)
GHGs/ emissions	"Consumption of beef, lamb and dairy must be cut by 20% by 2050 to reduce the methane emitted into the atmosphere by livestock." (Mirror, 28 July 2019)	"The NFU (National Farmers Union) says its three-part roadmap will produce quality, affordable food while tackling emissions." (BBC, 10 September 2019)
Weather	"[…] over the summer Amazon fires were three times more common in beef farming zones." (Guardian, 11 December 2019)	"An EU without livestock would […] mean increased fires […]" (Guardian, 15 October 2019)
Deforestation	"Production of agricultural commodities such as soy and palm oil is seen as a big driver of forest destruction." (Sky, 11 June 2019)	"He also argued there is a huge difference between the environmental impact of high-quality British beef for example, versus beef from an animal in a[n] […] area that has been deforested to make way for cattle." (Sky, 5 November 2019)
Land	"Clearing land to grow crops for animal feed also has a detrimental effect." (Sun, 8 August 2019)	"With 65 percent of British farmland only suitable for grassland, the most efficient way to turn this inedible grass into high-quality, nutritious protein is to graze livestock […]"(BBC, 22 November 2019)
Biodiversity	"Wildlife species are perishing…" (Guardian, 1 December)	"The campaign group says […] that livestock's contribution to biodiversity […] has been overlooked." (Guardian, 15 October 2019)
Water	"Slaughterhouses discharge wastewater contaminated with blood, oil and grease, and fats, which contains nitrogen and phosphorus pollution – pathogens – among other contaminants." (Guardian, 19 December 2019)	"A typical diet in Niger has the highest water footprint, researchers noted, mainly due to millet production […]" (MailOnline, 17 September 2019)
Sustainability	"Meat, milk and egg production has soared in the last 30 years […] This level of consumption is unsustainable and will see the Paris Agreement fail […]" (MailOnline, 16 December 2019)	"[…] the UK meat and dairy farming system is actually one of the most sustainable in the world […]" (Sky, 5 November 2019)
Reports: EAT-Lancet	"To protect the well-being of future generations, there needs to be a drastic reduction in the consumption of meat,	"[…] Brits have hit back claiming 'life wouldn't be worth living' without their daily dose of bacon." (MailOnline, 17 January

	poultry and eggs, the EAT-Lancet Commission says." (Sky, 17 January 2019)	2019)
Reports: IPCC	"Eating less meat could help save planet, a landmark United Nations report on climate change and land use suggests." (MailOnline, 8 August 2019)	"The new report on land by the Intergovernmental Panel on Climate Change (IPCC) shies away from the big issues and fails to properly represent the science." (Guardian, 8 August 2019)
Reports: Other	"The Committee on Climate Change's official recommendation to government is that a 20% cut in red meat and dairy is needed […]" (BBC, 11 October 2019)	"The committee on climate change, the government's official advisers, shy away from recommending big cuts in meat consumption. […] Both Sir David and Dr Springman[n] told Sky News the offsetting was not enough." (Sky, 15 May 2019)
Other	"[…] you could almost smell the steaming lakes of Barbie-pink toxic pig waste." (Guardian, 1 December 2019)	"Farmers are […] working with research to come up with viable solutions – such as reducing livestock enteric methane emissions through special feed supplements […]" (Guardian, 22 May 2019)

Section 5: Top three narratives and counter-narratives by news source

**Source**	**Top 3 most common narratives**			**Top 3 most common counter-narratives**		
**BBC**	GHGs (81.8%)	General (77.3%)	CC (63.6%)	Sustainability (36.4%)	General (31.8%)	GHGs (27.3%)
**BuzzFeed**	General, GHGs (100%)	-	-	-	-	-
**Guardian**	General (75.7%)	CC (59.5%)	GHGs (56.8%)	General (37.8%)	GHGs, Sustainability (29.7%)	Land (21.6%)
**LAD Bible**	General, GHGs (66.7%)	CC, Deforestation, Land, Water, Sustainability (33.3%)	-	Other (33.3%)	-	-
**MailOnline**	General (70.8%)	GHGs (62.5%)	CC (58.3%)	General, CC, GHGs, EAT-Lancet (16.7%)	Water, Sustainability, Other (4.2%)	-
**Mirror**	General (88.9%)	GHGs (77.8%)	CC (66.7%)	General, CC, GHGs, EAT-Lancet (11.1%)	-	-
**Sky News**	CC (78.6%)	General, GHGs (71.4%)	Sustainability (57.1%)	General (42.9%)	GHGs (35.7%)	CC (28.6%)
**Sun**	General (83.3%)	GHGs (66.7%)	Land, EAT-Lancet (50.0%)	EAT-Lancet (50.0%)	General, GHGs, Land, Sustainability (33.3%)	CC (16.7%)

Section 6: Sentiment distribution

a. Percentage distribution of sentiment (meat consumption) by news source

Source	Sentiment: meat consumption (% of articles per source)		
	Anti-meat	Pro-meat	Balanced
BBC	59.1%	0.0%	40.9%
BuzzFeed	0.0%	0.0%	100.0%
Guardian	64.9%	0.0%	35.1%
LAD Bible	66.7%	0.0%	33.3%
MailOnline	50.0%	16.7%	33.3%
Mirror	77.8%	0.0%	22.2%
Sky News	50.0%	7.1%	42.9%
Sun	50.0%	0.0%	50.0%

b. Percentage distribution of sentiment (meat industry) by news source

Sentiment: meat industry (% of articles per source)					
Source	Anti-meat (general)	Anti-meat (pro-sustainable)	*Total anti-meat*	Pro-meat	Balanced
BBC	4.5%	36.4%	*40.9%*	0.0%	59.1%
BuzzFeed	0.0%	0.0%	*0.0%*	0.0%	100.0%
Guardian	10.8%	35.1%	*45.9%*	0.0%	54.1%
LAD Bible	33.3%	0.0%	*33.3%*	0.0%	66.7%
MailOnline	12.5%	12.5%	*25.0%*	0.0%	75.0%
Mirror	11.1%	11.1%	*22.2%*	0.0%	77.8%
Sky News	7.1%	35.7%	*42.9%*	0.0%	57.1%
Sun	0.0%	33.3%	*33.3%*	0.0%	66.7%

Section 7: Distribution of articles about IPCC's SRCCL and EAT-Lancet

NB: BuzzFeed and LAD Bible are omitted from this table because they did not discuss the reports.

News source	# of meat-environment articles	# of report-related articles	% of report-related articles	# of report-related articles containing criticism	% of report-related articles containing criticism	% of meat-environment articles containing report-related criticism
**IPCC's SRCCL**						
BBC	22	3	13.6%	0	0.0%	0.0%
Guardian	37	8	21.6%	1	12.5%	2.7%
MailOnline	24	4	16.7%	0	0.0%	0.0%
Mirror	9	0	0.0%	0	0.0%	0.0%
Sky	14	1	7.1%	0	0.0%	0.0%
Sun	6	1	16.7%	0	0.0%	0.0%
**EAT-Lancet**						
BBC	22	4	18.2%	0	0.0%	0.0%
Guardian	37	6	16.2%	2	33.3%	5.4%
MailOnline	24	5	20.8%	4	80.0%	16.7%
Mirror	9	2	22.2%	1	50.0%	11.1%
Sky	14	1	7.1%	0	0.0%	7.1%
Sun	6	3	50.0%	3	100.0%	50.0%
